# Identifying critical drivers of innovation in pharmaceutical industry using TOPSIS method

**DOI:** 10.1016/j.mex.2022.101677

**Published:** 2022-04-04

**Authors:** Madhavi Damle, Bala Krishnamoorthy

**Affiliations:** a Symbiosis Institute of Digital and Telecom Management, Symbiosis International (Deemed University), Lavale, Pune, 412115, India; b Associate Dean (Accreditation), Professor and Area Chairperson, Strategy, SBM, Narsee Monjee Institute of Management Studies, Vile Parle, Mumbai, India

**Keywords:** Intellectual capital, Innovation indicators, Innovation system, Innovation rankings, Innovation index, Regional ranking, Priority indicators, TOPSIS, Technique for Order Preference by Similarity to an Ideal Solution

## Abstract

Insights in understanding critical growth drivers of innovation are essential for industrial development and economic growth for competitive advantage. This study identifies indicators from the world's major indexes and industry-level concerns. The indicators are mapped to these concerns using expert opinions and then treated mathematically using technique of order preference by similarity to an ideal solution (TOPSIS). This mapping ranks and identifies the most favorable indicators for several concerns. It, thus, identifies the critical role the indicators play for the drivers to the most effective advantage using the TOPSIS method as a comprehensive ranking of indicators effectively facilitates decision-making for estimated levels.

The method highlights are as follows:

• The most prevailing indicators for innovation are considered from the major innovation indexes for industries for mapping with concerns within the industry.

• Industry-specific concerns for the pharmaceutical industry are selected for the study.

• The mapping of indicators to concerns using expert opinion and using the TOPSIS method generated a matrix of the ranked indicators, aids in prioritizing resources and existing knowledge to resolve the concerns.

## Graphical abstract

**Figure:**
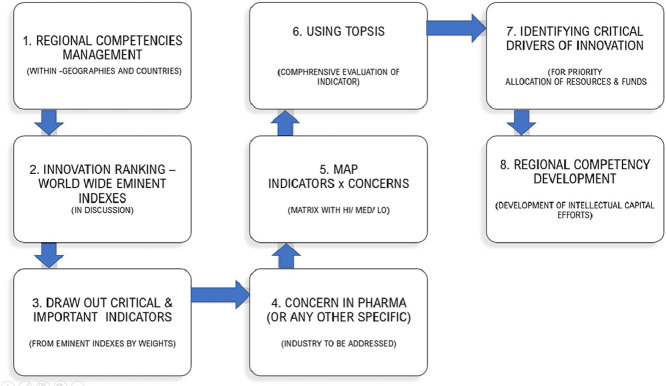

**Specifications Table**

**Table:** 

**Subject area**	Economics and finance
**More specific subject area**	*Business Management and Innovation; Regional Development of Innovation capabilities*
**Method name**	*TOPSIS*- Technique for Order Preference by Similarity to an Ideal Solution. (To rank Indicators for the concerns)
**Name and reference of original method**	Technique for Order Preference by Similarity to an Ideal Solution TOPSIS
**Resource availability**	*Resources are Research Articles.* [1], [2][1] *Yuan, L., Li, J., Li, R., Lu, X., & Wu, D. (2019). Mapping the evaluation results between quantitative metrics and meta-synthesis from experts’ judgements: evidence from the Supply Chain Management and Logistics journals ranking. Soft Computing, February.*https://doi.org/10.1007/s00500-019-03837-3[2] *Carayannis, E. G., Goletsis, Y., & Grigoroudis, E. (2018). Composite innovation metrics: MCDA and the Quadruple Innovation Helix framework. Technological Forecasting and Social Change, 131, 4–17.*https://doi.org/10.1016/j.techfore.2017.03.008

## Introduction

Innovation is one of the critical components for competitive advantage in businesses [34,35]. It is an output of science and technology that is engineered into practical applications. Innovation impacts the global economy [15], yet views about the process of innovation vary. Innovation is an abstract concept, an intangible entity, and complex in nature [27,51,53]. Thus, to explore it, grasping its essentials, and studying its management are important. With challenges in its implementation and its feasibility with diffusion and co-creation issues, for innovation to succeed is an intricate process [33,41,42]. Innovation as a process is cross-disciplinary and multifaceted [3,28,36] and its diffusion form an essential and crucial part, where insights would assist growth and success [49]. A strategic orientation, such as cost factors, productivity, resilience, and adaptation at business levels, is crucial for adaptability and readiness for innovation to succeed. Innovation is a strong driver of businesses and market forces dictate its acceptance. Innovation is enabled by human intellectual capital and other resources to create value [11,45,46,48,59]. In addition, the expected demand in the value proposition, managing the delivery of the value proposition to satisfy customers’ requirements and expectations is a challenge too [16,24].

Leading innovation indexes worldwide show indicators from macroeconomic factors which are direct contributions towards economies of the countries as aggregated from the business units. Indexes differ on their focus so the consideration of the indicators varies. In this study, while the indicators are recognized, it is important to differentiate these indicators and observe the formed perspectives in contributing to innovation studies accounting for their practical usage in real-life scenarios [16].

The primary objective for any index is about resources being in place for carrying out innovation activities. These resources include, but are not limited to, human capital, research, infrastructure sophistication, market superiority, knowledgebase, efficacy of technology delivery, creative outputs, etc. [25]. It ensures that innovation has the environment ready.

Some of the current studies also capture in the emerging paradigms in economies for dynamic factors such as propensity, which is a trajectory of growth towards resilient systems to innovate. The propensity in these study shows economies that are breaking out to be strong while some others that are weak or stagnant. Some significant areas are digital economy-driven parameters, an efficient system of knowledge and its diffusion, quality of governance, and entrepreneurial ventures.

In our study, we use 17 major indexes for innovation with their indicators for a comparison and to explore these indexes for their indicators. Section 2 discusses the highlights of significant innovation indexes and related indicators. Section 3 brings out issues and concerns related to the pharmaceutical industry. Section 4 discusses these relevant 32 indicators for their importance. Section 5 presents the methodology. Section 6 discusses the “technique of order preference by similarity to an ideal solution” (TOPSIS) for alleviating with mapping the ranks, identifies the most favorable indicators for several concerns. Section 7 is the discussion about the TOPSIS application framework. Section 8 is the critical analysis of Indicators and Industry concerns. Section 9 concludes the study.

For the identification of relevant indicators as enablers of innovation, the indicators are selected from major innovation indexes. Next, from the reviews from the literature, the concerns within the pharmaceutical industry are identified and the identified indicators are mapped to them. Moreover, the mapping is analyzed for critical drivers using TOPSIS. The framework will aid development of a comprehensive ranking of indicators needed to drive innovation. The ranking of indicators shows their contribution in the efforts required for each concern using the TOPSIS [58] method and the most efficient indicators provide the highest benefit to one or more concerns are sorted.

## Major innovation indexes

In this section, 17 significant indexes selected for this study are discussed. These indexes are rankings for various countries, and an inspection of the indicators helps in understanding the process and perspectives. Innovation effectiveness influences our understanding how innovation makes a difference to regions, nations, and industries. Table 1: List of Major Select Innovation Indexes lists the major indexes accepted by World Economic Forum, Issue March 2016.

**Table 1:** List of Major Select Innovation Indexes.

	EMINENT INNOVATION INDEXES			
	“Index	Source	Last Pub.	About the Index
1	Australian Innovation System Report 2017	Australian Government	2017	
2	Bloomberg Innovation Index	Bloomberg	2019	
3	Digital Evolution Index	The Fletcher School at Tufts University	2017	
4	Global Cleantech Innovation Index	Cleantech Group and World Wildlife Fund (WWF)	2017	Global Cleantech Innovation in clean technology
5	Global Entrepreneurship Monitor Global Report (GEM)	Babson College	2019	Applicability for Developing Countries
6	Global Intellectual Property Centre (GIPC) International IP Index	GIPC, US Chamber of Commerce	2019	
7	KAM Knowledge Index/Indicators	World Bank	2017	
8	Innovation Indicators – OECD Statistics	The Organization for Economic Co-operation and Development (OECD)	2018	To promote policies - economic and social well-being (1992, 1997, 2005, OSLO Manual Publications)
9	Innovation Union Scoreboard	European Commission	2018	
10	Innovation Indicators for Tomorrow	Alliance for Science and Technology Research in America (ASTRA)	2018	Applicability for Developing Countries
11	International Innovation Index	Boston Consulting Group (BCG) and the National Association of Manufacturers (NAM)	2018	
12	National Ranking of Science, Technology and Innovation 2013 (STI 2013)/ Pro-Cyclical Dynamics of STI Investment in Mexico	Foro Consultivo Cientifico y Tecnologico (FCCYT)	2018	Applicability for Developing Countries
13	UNESCO Institute for Statistics	United Nations Educational, Scientific and Cultural Organization (UNESCO)	2018	Applicability for Developing Countries
14	The Global Competitiveness Report 2015-2016	World Economic Forum	2018	Applicability for Developing Countries
15	The Global Innovation Index	INSEAD	2018	
16	The Massachusetts Innovation Economy Annual Index	Massachusetts Technology	2014	
17	World Development Indicators	The World Bank	2018	Applicability for Developing Countries”

Source: “Global Agenda Council on the Economics of Innovation, Evaluation of Leading Indicators of Innovation by World Economic Forum, Issue March 2016”

The studies vary on indexes as this depends on the perspectives for which they are undertaken. Comparing the trends becomes easy due to the periodical publication of indexes such as Global Competitiveness Report Index and European Commission's Innovation Union Scoreboard Index. Some publications, especially on methods or standards, such as “Alliance for Science and Technology Research in America” (ASTRA) [40], showing the future of innovation indicators, are one-time publications.

*“Australian Innovation System Report” 2017* explores innovation and entrepreneurship for new businesses like startups. Australian policymakers worry about whether Australia can innovate sufficiently to compete effectively in increasingly globalized markets for services and products. An aging population, slowdown in the global trade, global financial crisis, to name a few major ones, are hindrances Australia faces that counter its mainstream development. While, on the other hand, they are an agile nation, especially with growing firms that are innovation-active.

“Bloomberg Innovation Index” [11] publishes comparing measures for countries that focus on seven significant aspects of a country to be seen as innovative. This index measures the scores for research & development, presence of critical drivers such as high-tech companies, patient activity, human resources development, and others for the economy. Bloomberg uses databases from the “The World Bank” (WB), “World Intellectual Property Organization” (WIPO)- Conference Board, “Organization for Economic Cooperation & Development (OECD), and “United Nations Educational, Scientific and Cultural Organization” (UNESCO) as the primary data sources to assign rankings. One barrier that does not appear in its measurements is regulations and policies, which can be critical in innovation [10].

“Digital Evolution Index” (DEI), by Tufts University, is a set of parametric simulations that drive digital evolution from four defined drivers within the constraints and conditions of a digital market. The index considers drivers, which covers consumer income with demographics for their Internet usage, its focus is technology, infrastructure, for digital commerce taking into account for government policy and access to trade. Furthermore, consideration to Innovation creating startups with the ecology of competitive landscape is considered as well. The attractiveness of DEI index shows categories of countries as they emerge. These are stall out countries, watch out countries, stand out countries and break out countries in the digital economies. They have displayed a consistently impressive upward trajectory in improving their state of readiness and are well poised to break into the Stand Out ranks in the years to come” [14]

“Global Entrepreneurship Monitor Global Report” (GEM) by the Babson College, discusses the entrepreneurial thought and action (ETA). The indicators are an experiment on real-life parameters and focus on the opportunity window for a value proposition for a business launch. Attributes of prominence used in this index are social, environmental, economic responsibility, and sustainability (SEERS), and value creations. The focus of GEM is on three indicators: total early-stage entrepreneurial activity (TEA), entrepreneurial employee activity (EEA) and social entrepreneurial activity (SEA) [6]

“Global Intellectual Property Center” (GIPC) International 2007, GIPC works for the protection of intellectual property rights and aids in innovative solutions with the US. Their perspective is for competitiveness and economic growth. The primary task of GIPC is protection of an innovation until a successful launch. GIPC protects and preserves the intellectual properties of 38 economies and assists them in economic growth and innovation [23].

“Knowledge Economic Index” (KEI) by the World Bank, follows the knowledge assessment methodology (KAM) for a knowledge-based economy's rapid development due to drivers such as digital technologies. KEI monitors four essential paradigms: “(i) Rapid development of Pharmaceutical, (ii) Acceleration of technological progress, (iii) Global competition, (iv) Evolution of consumers’ preferences.” The essential monitoring is for the usage of scarce resources for their utility and for how connected are the resources for the various sectors of the economy and performance as measured against a global benchmark. This is a challenge faced by countries, and the perspectives of this index help in gaining information to resolve this challenge through annual research initiated by the World Bank called “Knowledge for Development (K4D).” KEI is for the analysis of performance for the readiness of economic model based on knowledge. The knowledge economy estimates use seven clusters of performance with 148 indicators [18]

Innovation Indicators, OECD Statistics, has for its mission, “Organization for Economic Co-operation and Development” (OECD), to promote policies that will improve people's economic and social well-being worldwide. The OECD provides a forum where governments can work together to share experiences and seek solutions to common problems.” Moreover, policies are designed to improve people's lives. OECD provides a platform so that governments can seek assistance and work together on common problems. The objectives are establishing economic enhancements and drivers of social and environmental change. It helps to understand, estimate, and analyze future trends [38]. OECD oversees the functional part of National Experts on Science & Technology Indicators (NESTI).

“Innovation Union Scoreboard”, EU 2018 is an index of the comparative status of the members of the European Union. It's perspective is the productivity issues concerning innovations, assessment for their competencies, and the areas for improvement.

UNESCO demonstrates with statistical capabilities the progress in 1999. The UNESCO Regional Office studied other relevant disciplines such as science and technology, environmental studies, oceans, climate studies, biodiversity, and disaster studies. These disciplines do imply regional development [50] .

Investment in Science, Technology, and Innovation (STI) is a forum that focuses on economic and social developmental studies, where R&D infrastructure activities are also focused on development. STI policies developed by this forum are also advised and guided by UNESCO.

“Global Competitiveness Report” (GCR) by the World Economic Forum is a yearly publication that inspects about 140 plus economies for insight and does a country wise analysis. The “Global Competitiveness Index” 4.0 integrates both the macroeconomic and the microeconomic aspects of competitiveness into a single index [57].

“The Global Innovation Index” (GII) [17], with the partnership of the Cornell University, INSEAD, and the World Intellectual Property Organization (WIPO), measures innovation with two parts: input sub-index and output sub-index [54].

The major indexes of innovation capture several aspects with few surveys based on the OECD norms, and in the indexes listed above, the survey reports are also considered for the indicators. Differentiated studies have also been carried out for sectoral indices, social indexes, trends, etc. These studies do not have generalized parameters but they investigate the phenomenon. For the sectorial, some studies are carried out by the Institute for Defense Analyses (IDA), Science & Technology Policy Institute and National Endowment for Science, Technology, and the Arts (NESTA) with very different perspectives. There are also other studies that examine innovation patterns and their understanding, with trends such as resources utilized for innovation, innovative youth force, everyday innovation. On the topic of human resource with socioeconomic and intellectual linkages to the human capital, creates an intellectual value chain.

In the next section, the major indicators and their repeated occurrence in multiple indexes shows their importance, so its consideration for monitoring is important.

## Relevant indicators as enablers of innovation

Indicators that repeat in different studies seem to be the ones that emerge as significant. Those listed in Table 2: Relevant indicators mapped for occurrence matrix, are considered to be the significant ones. There are very few indicators that measure the outcomes for the innovation and its success and we follow the outcomes given by OECD [37,38,52] and World Economic Forum (WEF) [56]. This paper analyses five levels of innovation capability in the following parameters: first, in the organizational structure and capability; second, in the process innovation; third, in the service innovation; fourth, in the product innovation capability; and the fifth in understanding the markets for their marketing innovation capability to provide overall information for the national innovation capability index [43,55]

**Table 2:** Innovation indexes and critical & relevant indicators - Matrix for calculation of Weights.

Source: World Economic Forum: Global Agenda Council on the Economics of Innovation Evaluation of Leading Indicators of Innovation (March 2016)

**Table 3:** Major Indicators and Pharmaceutical industry concerns are associated and mapping – The Matrix. (An illustration).

	Indicators Affecting the ICTIndustry in the Following Areas					
		Local and International Regulatory Role and Legal Risk	Operational Complexity and Susceptibility	Talent and Skills	Business Model and Adaptability	Digital Vulnerability and Rapid Technology Advancement
1	Education Level of Population	Low	High	High	Medium	High
2	R&D Expenditures	Low	Low	Low	High	High
3	Patent Activity	Low	High	High	High	High
4	New Products and Services Introduced	Medium	High	High	High	High
5	Venture Capital	Medium	High	Medium	High	Medium
6	Exports of Knowledge-Intensive Products and Services	High	High	Medium	High	Medium
7	Foreign Direct Investment (FDI) Inflows	Medium	High	Medium	High	Medium
8	Small and Medium-Sized Enterprises Collaboration	High	High	Medium	High	Medium
9	Public Policies	High	High	Low	Low	Low
10	Scientific Publications	Low	Low	High	High	High
11	Labour or Workforce Productivity	Low	Low	High	Medium	Medium
12	Organizational and/or Marketing Improvement	Low	High	Medium	High	Low
13	R&D Personnel (Human Resources)	Low	High	High	High	High
14	Employment in Knowledge-Intensive Activities (“Mfg &Ser")	Low	High	High	High	High
15	The Small Business Innovation Research	Low	High	Low	Low	Low
16	Technology Transfer	Low	High	High	High	High
17	International Cooperation in Patents	High	Medium	Medium	Medium	High
18	Sales of New-to-Market and New-to-Firm Innovations	Medium	Medium	Medium	High	Medium
19	Public Attitudes and Sources of Information	High	Low	Low	Low	Low
20	Intellectual Property (IP) Protection	High	Medium	Low	Medium	Low
21	Royalty and License Fees Payments and Receipts	Low	Medium	Low	Medium	Medium
22	Core Characteristics of Entrepreneurial People	Low	High	Medium	High	Medium
23	High-Tech Density	Low	Medium	High	High	High
24	Education Policy	High	Low	Low	Medium	Medium
25	Number of Patents in Force	Medium	Medium	Medium	Medium	High
26	Entrepreneurial Culture/Societal Attitudes	Low	Medium	Medium	High	Low
27	Infrastructure	Medium	High	Low	High	High
28	Initial Public Offerings (IPO)	Medium	Low	Low	High	Medium
29	Inhibitors to Innovation	High	High	High	Medium	Medium
30	Knowledge-Sharing and Collaboration Opportunities	Low	Medium	Medium	High	Medium
31	Science, Technology and Innovation (STI) Literacy	Low	High	High	High	High
32	Company Proximity to Others	Low	Medium	Medium	Low	Medium

Innovation as an asset in business is visible as intellectual property, royalty fees income, license fees for consultation

Certain organizational cultures are more aligned towards free thinking and are dynamic in allowing innovation. Such practices build up and develop innovation cultures as an essential part of organizational practices [21,22,30]. Furthermore, the people engaged in networking and collaborating will have a greater potential for innovation and will understand the dynamic linkages involved [12,13]. DEI includes dynamic indicators that show the development a region in time. DEI depicts the trends of progress in the economics of countries for developmental studies of innovation.

Global Entrepreneurship Monitor Report shows the development for innovation capabilities, which is the value created in a system. KAM Knowledge Index includes the components of knowledge management, showing that knowledge dissemination for innovation is important. KAM by World Bank has worked in different areas to create detailed reports for the betterment of the world. The EU scoreboard measures the progress and captures the capabilities of member states and firms therein.

Innovation is a phenomenon that requires unique capabilities within the organization. One of the capabilities is knowledge management as an asset. Knowledge and its translation in developing new knowledge are complex phenomena that involve generation of an idea to a successful innovation [3].

Specific measures are predominantly used in many indexes and have definite properties that are necessary to study innovation. These are important parts of the study [47].

Once an innovation is registered, it is protected as intellectual property (IP) for the company, and it may become a source of revenue stream. Intellectual property may be accounted for its performance. The OECD lays down the norms for development measures in its reports for innovation

UNESCO has aided the developing nations in Latin America and Caribbean Islands and neighboring countries for research and development and infrastructure improvement, which are critical for development with necessary primary conditions met .

Table 2: Relevant indicators mapped for occurrence matrix, is indicators as a matrix, by weights by occurrences. Here 32 frequently occurring indicators are seen emerging as essential contributors as essential ones [57] of which 27 are chosen. Those indicators with weights of more than one is considered in the study, of which the top ones are as follows.

1) With the weight of 14, “Education level of the Population” is emerges as the highest and a strong indicator
2) At the second and third position with the weight of 12 are indicators “R&D Expenditures” and “Patient Activity.”
3) With the weights of 8 and 7, there are four indicators: “New Products and Services Introduced,” “Exports of Knowledge-Intensive Products and Services,” “Venture Capital” for funding, and “Foreign Direct Investment (FDI) Inflows.”
4) There are six indicators with the weights of 6 and 5: “Scientific Publications,” “Small and Medium-Sized Enterprises Collaboration,” “Public Policies,” “Labor or Workforce Productivity,” “Organizational or Marketing Improvement” and “R&D Personnel (Human Resources)”.

These are the indicators stated here have more weight that is they appear in several indexes, hence considered as important indicators.

## Pharmaceutical industry: specific issues and concerns

Several issues affect the drivers for innovation in the pharmaceutical industry. Following are the primary concerns and issues that matter for growth and development and are considered for this study [26,32].

### Local and international regulatory role and legal risk

All entities in the pharmaceutical industry need a common platform of regulation and norms accepted norms that are followed globally. This is a significant step towards unification of a common platform to operate in the pharmaceutical industry and the requirements of the local governments to protect all stakeholders with policy implementation such as anti-trust law, political stability, neutrality, and cybersecurity, as these are major areas of impact in businesses and organizations, and can have a direct bearing on strategies, commercial arrangements, and operational decisions.

Patents and licenses are significant barriers in managing a business [4], as the performance of the company depends on its innovation output; especially high-complexity and high intensity is needed to develop and manage innovation capability, learning, and development.

Patient-driven drug development is a challenging and long-drawn process in pharmaceuticals. Development in a pharmaceutical company is an emerging research area and patients and their advocates must engage in drug discovery. Possibilities of early-stage drug research that shows promise of success have distinct advantages, and these will also gain support from regulatory bodies. The clinical and scientific trials are carried out due to the pressures from advocacy groups and patients’ influence for which the therapies that are developed, by financing promising treatments, that otherwise would not secure funding. Though some critics of patient-driven drug development worry about the ethical and scientific implications of this new approach to research, it also has several advantages over the current system. These significant challenges at the cutting edge of drug discovery and legal systems may need a resolution from process-driven outcomes [20].

### Operational complexity and susceptibility

Companies operating in the pharmaceutical industry have to deal with complex operations that are susceptible to market demands and operations within the company. Significant factors such as human resources, other resources, processes, interdependency, and security are regulated while these are significant for capability development and sustained business operations. The foundation for the management of complex systems is a challenge. The complexity is very high in this regulated industry, and the firms developing new drugs face challenges of handling complex systems, and negotiating the discoveries towards undisputed successful outcomes for market acceptance is difficult [8]. Raw material availability, commercialization, delayed product introduction until successful development of a stable version make the journey riddled with major strategic decisions, covering the time to develop efforts, resources deployed, and financially feasibility [9].

### Talent and skills

The pharmaceutical sector demands a high quality of human intellectual capital and sound systems of operations. A skilled human resource is a scarce resource, which is a critical success factor in operations. The intervention from the regulatory authorities must meet the market demands and further facilitate employment for capability built up and capacity mapping of human resources employed. Furthermore, development of skills and capabilities is required such that innovative may be taken up well, thus managing this workforce responsibly reshapes talent management paradigms [31].

Talent management is essential for businesses, especially in the pharmaceutical industry in the Indian context which is yet to mature but is lucrative. A steady growth is seen globally in international organizations, but talent acquisition and management in India is yet to meet the international standards. Talent management is a critical part of business strategy as a significant driver of innovation and not just a simple function of human resource management. Engaging senior leaders at strategic levels for talent management is the need of the hour in light of intense competitive forces for businesses [44].

Business Model and Adaptability

Pharmaceutical industry is continuously upscaling in technological innovations, and this sets a precedence in the sector demands for continuous innovation and agility for being adaptable to different skill sets and capabilities of the workforce, business strategy deployed, and adaption of processes in operations in operations. The threat from new market entrants and an increasing demand drive firms to not only innovate but to reevaluate their operational configurations and business models as well.

It is a compelling argument, made by Lazonick, who states that the major pharmaceutical companies manage through high prices for the drugs that are sold, the companies maximize the shareholder value so that the profits are used for buybacks of their corporate stock. These may account for controlling their stock prices while providing redemptions and compensation that reward the key partners for performance. Thus, there is unrest in how models of the capital structure may change and affect the operational levels [29].

### Digital advancement and vulnerability

The pharmaceutical sector, with its challenges, has its infrastructures and processes riding on technologies and its development. Scaling of technological progress for consumer service and expectations for its alignment with business capability for competition is necessary. An investment in IT infrastructure and design processes enhances the delivery. The enhancements in technology and its implementation challenges bring in information security risks.

The pharmaceutical industry in the fourth industrial revolution with a breakthrough from digital revolution has taken an unprecedented step towards advancement. These transform pharmacies into digital pharmacies, with multiple channels of diagnosis and delivery, telemedicine care cycle, and definitively modifying the pharmacotherapeutic treatment of patients. Several changes are observed due to provisions such as flexible dosages, drug discovery, multi-active pharmaceutical ingredients. This also brings in its challenges for all stakeholders to align towards the progression of business requirements. The technological leap has been seen in the pharmaceutical sector and, according to the most optimistic prognostic, it will develop further with advances in applied technologies in the pharmaceutical industry [5].

## Methodology

In this study, major indexes and their indicators are considered. The indicators are mapped to the industry concerns and experts determine the level of impact of the indicators on concerns within the pharmaceutical industry using TOPSIS analysis. using TOPSIS analysis.

TOPSIS maps for ranking the outcome from the opinions of intellectual knowledge workers [58].

The steps for the analysis are as follows.

1 Indicators as enablers affecting the concerns of pharmaceutical industry and are mapped with levels in Table 2: Major Indicators and Pharmaceutical industry concerns are associated and mapping – The Matrix. (An illustration).
2 The concerns of pharmaceutical industry are listed in columns while the indicators are in rows.
3 Expert respondents, who are professionals or intellectual knowledge workers,
4 The respondents mark each matrix with the impact levels of the indicators as high, medium, or low, for each concern. These matrices are collated and taken up for analysis.
5 The data is analyzed through the scores collected for ten responses to demonstrate the method. Further, as shown in Table 2: Major Indicators and Pharmaceutical industry concerns are associated and mapping – The Matrix. (An illustration), indicators mapped to the concerns of the pharmaceutical industry are taken up for analysis.
6 TOPSIS is employed by first setting up the evaluation Matrix X, which consists of *m* indicators and *n* concerns of the pharmaceutical industry.
7 Matrix X is normalized to Matrix R to obtain a weighted normalized matrix.
8 The alternatives then are calculated such that for the most concerned issue the indicator has the highest weight.

## Indication selection development

In this study, the focus is on developing a comprehensive selection of indicators for implementation of the method by prioritizing optimization of critical innovation indicators. The method used is mapping the industry's concerns against critical indicators. A matrix is formed from this mapping, and is used for analysis employing TOPSIS.

TOPSIS APPLICATION FRAMEWORK

### The process of application

The following steps are used in developing the framework:

1) The mapping is *m* x *n*, that is, indicators in the rows are mapped with concerns in the columns (Refer Table 2: Major Indicators and Pharmaceutical industry concerns are associated and mapping – The Matrix. (An illustration)) and each cell is populated as either high, medium, or low by expert respondents.
2) The data was collected from 28 respondents for each indicator through assigning of a level of impact of the indicators for each concern.
3) The evaluation matrix consists of *m* indicators and *n* concerns for the industry.

The method used here is TOPSIS, used for multiple criteria for decision-making problems. The basis of the selection depends on the alternatives closest to an ideal possible solution in terms of Euclidian distance of alternatives. The optimized solution in terms of value or benefits accrued will fit while optimizing each criterion. TOPSIS is a valuable technique for ranking several possible alternatives according to their closeness to the ideal solution. We have calculated these values for the most concerned as the highest weight in our study. The actual process is the creation of a decision matrix of *n* alternatives, *m* criteria, and a set of weights for the criteria. Next, an outcome expressed in a non-numerical way, quantified through appropriate scaling technique, is checked. Finally, the preferred alternative should have the shortest geometric distance from the positive ideal solution (PIS) and the longest geometric distance from the negative ideal solution (NIS).

### Method

Innovation indexes analyze accepted measures of indicators and capture innovation activities well enough for the macro environment. A comparative scale is for a broad overview of the economy and industry. Indexes can point out different regions as weak performers and even nonperformers through a comparison.

**Fig. 1:**
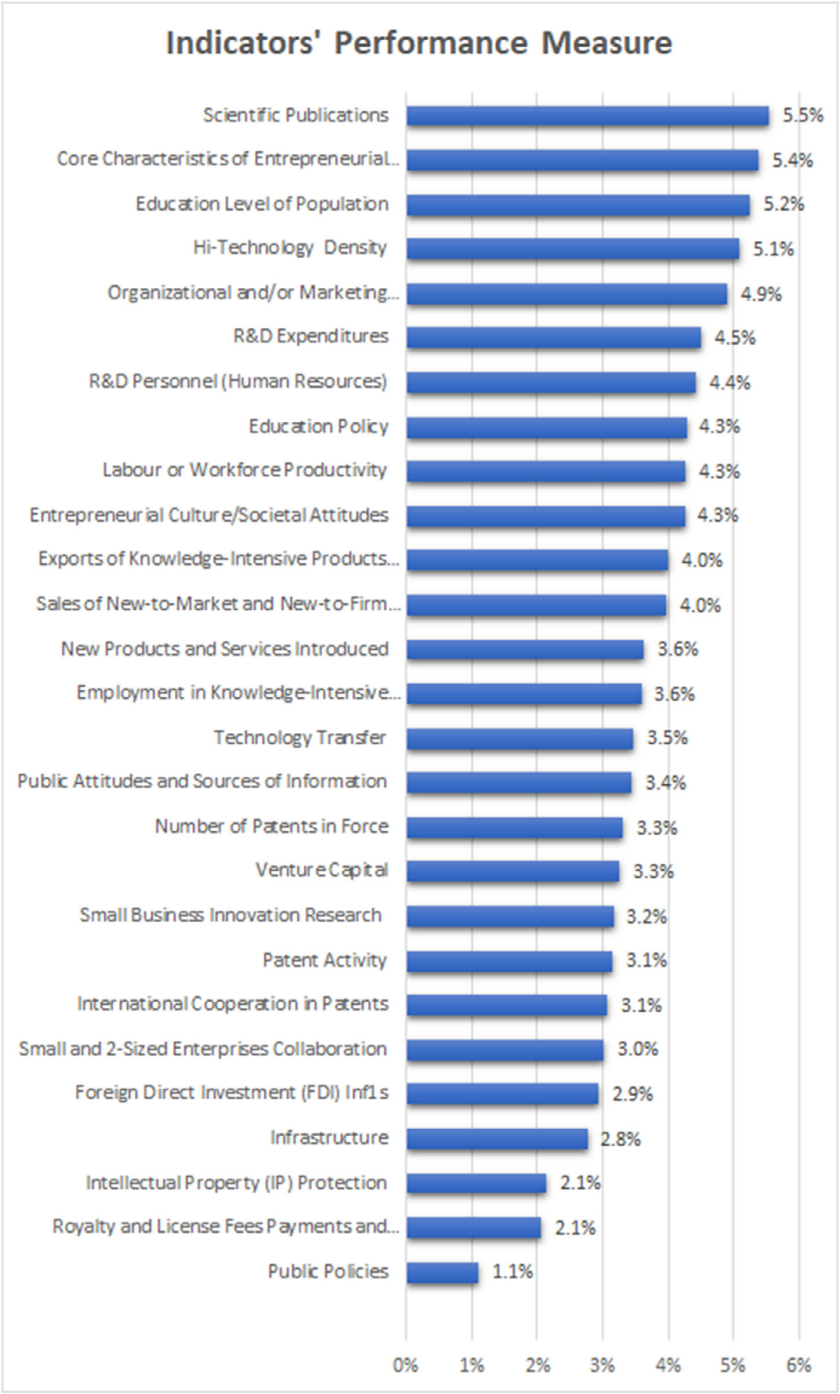
Selection of Indicators with Prioritizing Indicator using TOPSIS.

The analysis is done in two parts: scores for the indicators and TOPSIS analysis. The scores on the data have been plotted in the Figure 1: Selection of Indicators with Prioritizing Indicator using TOPSIS, with the received score calculated for each indicator.

TOPSIS analysis has been carried out with the following steps.

**Step 1**

Calculation of scores with High, Medium, and Low levels for each concern. High is considered 3, Medium as 2, and Low as 1. These are added to obtain the final score for each indicator (total of 27 indicators) mapped with each concern (total of five concerns). This forms a matrix of 27 × 5. Each cell is now a data point for calculation. Thus, the matrix is as follows.

(1) $$R_{mxn}\;=\;{\left(x_{ij}\right)}_{mxn}$$

where i= 27 and j=5. With each element i=1 to 27 and j=1 to 5

The matrix as a normalized matrix is constructed using Eq 2.

(2) $$r_{ij}=\frac{x_{ij}}{\sqrt{\sum_{k=1}^{m}x_{kj}^{2}}}$$

where x is the element with the normalized matrix and w is the weight.

**Step 2**

To calculate the weighted normalized matrix for each concern and criterion, weighted normalized decision matrix was created with each element multiplied by the weight. Since there are five concerns, the weight is 20% each.

(3) $$t_{ij}=\;r_{ij}\;.\;w_{j}$$

**Step 3**

To determine the worst and the best alternatives for the concerns, we select which indicators are under observation. Here the idea is to capture the ideal worst and the ideal best value.

(4) $$\left(A_{worst}\right)\;=\;\left\{\begin{array}{c} \left[\max(t_{ij}|i=1,2,\ldots ,m)|j\;J_{-}\right),\\ \left[\min\left(t_{ij}|i=1,2,\ldots ,m\right)|j\;J_{+}\right) \end{array}\right\}\equiv \left\{t_{wj}|j=1,\ldots ,n\right\}$$

(5) $$\left(A_{best}\right)\;=\;\left\{\begin{array}{c} \left[\min(t_{ij}|i=1,2,\ldots ,m)|j\;J_{-}\right),\\ \left[\max\left(t_{ij}|i=1,2,\ldots ,m\right)|j\;J_{+}\right) \end{array}\right\}\equiv \left\{t_{bj}|j=1,\ldots ,n\right\}$$

Equations 6 and 7 show the best and the worst values associated with the indicator.

(6) $$J_{+}=\left\{j=1,2,\ldots .,n|j\right\}\;associated\;with\;critera\;having\;positive\;impact$$

(7) $$J_{-}=\left\{j=1,2,\ldots .,n|j\right\}\;associated\;with\;critera\;having\;negative\;impact$$

**Step 4**

For this study, the areas of concern are listed in Table 4: Ideal best situation for concerns in the pharmaceutical industry. The calculation of distance L^2^ between the target alternative “i” and the worst condition A_w_ and the best condition A_b_ is as follows.

(8) $$d_{iw}=\;\sqrt{\sum_{j=1}^{n}{\left(t_{ij}-t_{wj}\right)}^{2}},\text{Where}i=1,2,\ldots ,m.$$

(9) $$d_{ib}=\;\sqrt{\sum_{j=1}^{n}{\left(t_{ij}-t_{bj}\right)}^{2}},\text{Where}i=1,2,\ldots ,m.$$

Here, the distances “d” is the worst and best conditions calculations in equations 8 and 9. Next, we calculate the similarities among the worst conditions.

**Table 4:** Ideal best situation for concerns in the pharmaceutical industry.

Concerns from the ICT industry	Ideal Best
Local and International Regulatory Role and Legal Risk	Low
Operational Complexity and Susceptibility	Low
Talent and Skills	High
Business Model and Adaptability	High
Digital Vulnerability and Rapid Technology Advancement	High

**Step 5**

These are calculated as follows the alternative solution has the best outcome, while S*_iw_* =0 is considered the worst.

(10) $$S_{\mathrm{iw}}=\;\frac{d_{\mathrm{iw}}}{\left(d_{\mathrm{iw}}+d_{\mathrm{ib}}\right)}\;\mathrm{Where}\;0<\;S_{\mathrm{iw}}<1\;\mathrm{for}\;i=1,\;2,\;\ldots ,\;m.$$

Here S*_iw_* =1 if and only if the alternative solution has the best outcome, while Siw =0 is considered the worst. Finally, the performance ranking alternatives are selected from S*_iw_* where (i = 1, 2, …, m).

The framework developed with the priority settings is seen in the further analysis for the combined level priority explained below. A combined level for high priority, medium priority, and low priority responses has been mapped from the responses. In this study, we have used only three grades for developing the critical concerns that can be mapped to five or more levels. Many other indicators come second to the critical indicators, which show the highest levels of priority at the top performance parameters of TOPSIS.

## Analysis and discussion

It can be seen that the indicators which have a high setting of priority on concerns for the pharmaceutical industry are

• The first group of enablers have the highest priority. These indicators are significant drivers that will alleviate several concerns. They include (i) Scientific publications, (ii) Core characteristics of entrepreneurs, (iii) Education level of the population, (iv) Hi-technology density. These are taken for about 5% performance measure.
• The second group of enablers are (i) Organization and marketing improvement (ii) R&D expenditures, (iii) R&D personnel (Human Resources), (iii) Education Policy (iv) New to the market and new to the firm
• From the indicators selected, the remaining fall below 4%, and these are the remaining 15 indicators of the 27 indicators considered for analysis in this study.

This study focused on the indicators needed by most of the concerns to move upwards in levels. The framework helps identify the indicators that can drive the internal controls for possible best outcomes for several industry-specific concerns, which are external pressures that the firm can address. The concerns for the pharmaceutical industry are broader and the indicators give an insight for addressing its issues and the level required for its development. However, the framework can be applied at the organization level or industries or at regional levels for a further study of critical indicators, which have been substantiated from major indexes as the indexes’ focus has been on the indicators’ prominence and perspective. The present study gives the current status of enablers in the industry and allocates priorities such that the concerns are addressed optimally for the readiness to compete.

The critical factors have an economic impact on global competitiveness [39], employment, management of intellectual property rights, and outcomes [7]. The indicators with medium-level priority may be addressed as secondary and having a low impact as they are either lagging indicators or have long-term prospects for outcomes.

## Conclusion

The study prioritized various innovation indicators for planning or decision-making. By appropriate allocation checks for optimization of efforts, the framework shows the priority in which the resources should be allocated. The studied indicators can be used for an enhanced and improved resilience building of innovation competency [1,2,19].

This study focused on the optimization of prevailing capabilities as indicators. The proposed framework identified critical indicators as drivers, taken from major innovation indexes, which were mapped to the vital concerns recognized in the pharmaceutical industry and were further analyzed using TOPSIS to identify optimal resource allocation for the indicators to yield the most efficient outcomes towards industry readiness of innovation. The framework aids in the development of a comprehensive ranking, from maximum to minimum, of the indicators that are needed to drive efforts in innovation analyzed critically. These estimates assist in identifying the critical drivers so that leadership and policymakers can make decisions and take strategic calls.

## Appendix Supplementary materials

Image, application 1
